# Chemotherapy versus chemoradiotherapy for FIGO stages IB1 and IIA1 cervical squamous cancer patients with lymphovascular space invasion: a retrospective study

**DOI:** 10.1186/s12885-022-09309-6

**Published:** 2022-02-23

**Authors:** Hao Zhang, Rao Yu, Lan Zhang, Rong Wang, Lin Xiao

**Affiliations:** Department of Gynecology, The First Affiliated Hospital of Chongqing Medical University, Chongqing, 400016 China

**Keywords:** Uterine cervical neoplasms, Risk factor, Chemoradiotherapy, Chemotherapy, Adjuvant, Progression-free survival, Survival analysis

## Abstract

**Purpose:**

To evaluate the impact of different adjuvant therapy on IB1 and IIA1 stage cervical squamous cell cancer patients with lymphovascular space invasion. It also aimed to analyze the relationship between lymphovascular space invasion and other clinical pathological characteristics on IB1 and IIA1 stage cervical squamous cell cancer patients.

**Methods:**

This retrospective observational study collected data of FIGO stages IB1 and IIA1 squamous cervical cancer patients at the First Affiliated Hospital of Chongqing Medical University between 2014 and 2018. A correlation analysis between lymphovascular space invasion and other clinical or pathological factors was conducted. Prognosis analysis of patients with lymphovascular space invasion were performed to assess associations between clinical-pathological characteristics and survival.

**Results:**

A total of 357 women were identified including 110 (30.8%) with lymphovascular space invasion, 247 (69.2%) without lymphovascular space invasion. Both middle 1/3 cervical stromal invasion (*p* = 0.000) and deep 1/3 cervical stromal invasion (*p* = 0.000) were independently associated with lymphovascular space invasion. Among lymphovascular space involved women, tumor differentiation (*P* = 0.001) and postoperative therapy (*P* = 0.036) had a significant influence on disease recurrence. Multivariate analysis showed that lymph node metastasis (*P* = 0.017), poorer tumor differentiation (*P* = 0.036) and postoperative chemotherapy alone (*P* = 0.021) can increase the risk of tumor relapse.

**Conclusion:**

Our study suggested that the presence of deep stromal invasion independently increases the risk of lymphovascular space invasion. Compared with chemotherapy, chemoradiotherapy seems to improve progression-free survival in squamous cervical cancer patients with lymphovascular space invasion.

## Introduction

Cervical cancer, being the fourth most frequent malignant tumor among women, is also one of the leading causes of female death. In 2020, it was estimated that there were 604,000 new cases of cervical cancer and 342,000 deaths worldwide [1]. Approximately 80% of all cervical cancer are squamous cell cancer, while adenocarcinoma and other pathological type makes up the rest. With the popularization of the screening test, more and more patients are diagnosed with cervical cancer in an early stage. Surgery is the preferred modality for the treatment of early invasive cervical cancer (FIGO stage 2009 IB1, IIA1), which usually consists of a type C radical hysterectomy with pelvic lymphadenectomy [2]. Following radical hysterectomy, postoperative treatment is indicated for patients with adverse pathologic factors.

Among various pathologic factors, lymph node metastasis, parametrial involvement and positive surgical margins are considered high-risk factors for the poor prognosis of cervical cancer, while lymphovascular space invasion (LVSI), deep stromal invasion (DSI) and tumor diameter greater than 4 cm are considered as intermediate risk factors [2, 3]. Lymphovascular space invasion is the presence of cancer cell clusters inside endothelium-lined channels of uterine specimens [4]. LVSI is considered to be an important risk factor which portends poor prognosis in patients with low-risk endometrial cancer, and it is also found to be associated with lymph node metastasis [4–10]. However, there’s only limited data on the prognosis of cervical cancer patients with LVSI, with very heterogeneous results. It is generally agreed that LVSI is related to poor prognosis of cervical cancer, while there is still controversy on whether it is an independent prognostic factor and its association to other pathological risk factors.

Generally, for patients with high-risk individuals, postoperative radiotherapy plus concurrent platinum-based chemotherapy is recommended. However, for patients with intermediate risk factors, postoperative therapeutic regimen still remains controversial.

There are guidelines recommended that pelvic radiotherapy with (or without) concurrent platinum-containing chemotherapy should be offered to patients with combination of any two or three of the intermediate risk factors. This recommendation is based on a prospective randomized study (GOG #92), while in 2006 the follow-up of the same study revealed that even if adjuvant radiotherapy significantly reduces the risk of recurrence and prolongs progression-free survival (PFS) in women with Stage IB cervical cancer, the overall survival (OS) did not change significantly (p 0.074) [11, 12]. Therefore, some studies have attempted to add chemotherapy to adjuvant radiotherapy, and found that compared with radiotherapy alone, chemoradiotherapy might be more effective as an adjuvant therapy for intermediate-risk early cervical cancer [13–17]. On the contrary, a randomized phase III trial revealed that chemoradiotherapy is not superior to radiotherapy alone for early stage cervical cancer patients with intermediate-risk factor [18].

The present study was aimed to evaluate the impact of different adjuvant therapy on IB1 and IIA1 stage cervical squamous cell cancer patients with LVSI as well as to analyze the relationship between LVSI and other clinical pathological characteristics.

## Materials and methods

Postoperative patients who had early-stage cervical cancer at the First Affiliated Hospital of Chongqing Medical University in recent years were retrospectively analyzed. Medical records were obtained with informed consent of all patients. The inclusion criteria were: (1) diagnosed with cervical cancer at the First Affiliated Hospital of Chongqing Medical University from February 1, 2015 to December 31, 2018; (2) FIGO stage (2009) IB1 or IIA1; (3) with definite histological diagnosis of squamous cell cancer; (4) has received a radical hysterectomy with pelvic lymphadenectomy; (5) with or without lymphovascular space invasion and other risk factors. Exclusion criteria including: (1) has received surgery or chemotherapy or radiotherapy for cervical cancer at other hospital before the consultation at the First Affiliated Hospital of Chongqing Medical University;(2) has received neo-adjuvant therapy; (3) with any component of adenocarcinoma, neuroendocrine carcinoma or other differentiation within the tumor; (4) accompanied by any other kind of malignancy; (5) absence of follow-up data.

The baseline information of included patients was retrieved from patient files, including age, BMI, FIGO stage, approach of surgery, value of squamous cell carcinoma antigen (SCC, ng/ml), clinical tumor size. Each patient has received a pathological examination after the surgery, data as follows were collected: pathological type, tumor differentiation grade, lymphovascular space invasion, deep stromal invasion, lymph nodes involvement, parametrial involvement, surgical margin involvement and expression of P16. All the surgeries were performed by qualified and experienced surgeon. The determination of postoperative treatment was based on practitioners’ assessment of the condition as well as patient’s intention to treatment. For patients received chemotherapy, the cisplatin-based plus paclitaxel regimen was given every 3 weeks, consisting of paclitaxel 135 mg/m^2^ and cisplatin 60 mg/m^2^ for 2–6 cycles. For patients received radiotherapy, intensity-modulated pelvic radiotherapy (IMRT) with a dose of 50 Gy in 25-28 fractions was prescribed. In cases with positive lymph node, regional radiation dose can be increased upto 60 Gy. Prognosis information such as recurrence, metastasis, and death were obtained from medical record or telephone interview. All patients were followed up until December, 2020. The primary endpoints of the present study were overall survival (OS) and progression-free survival (PFS). OS was defined as the time from surgery to death of any reason or the most recent follow-up, and PFS was define as the time from surgery to recurrence or metastasis [19].

All patients were enrolled in a correlation analysis between LVSI and other clinical or pathological factors. Patients were grouped according to the presence of LVSI. Chi-square test and t-test was used to compare demographic and clinical-pathological characteristics between LVSI-positive (LVSI+) and LVSI-negative (LVSI-) individuals. Binary Logistic regression was used for multivariate analysis. Patients with LVSI+ were grouped according to different type of adjuvant therapy (observation, chemotherapy alone (CT), radiotherapy alone (RT), chemoradiotherapy (CRT)). Patients with observation and radiotherapy alone were not included in the comparison because of limited data (6 patients of observation and none of radiotherapy alone). Clinical and pathological characteristics of included individuals were compared using chi-square test or Fisher exact test for frequencies and student t-test for continuous variables. The prognosis information such as PFS and OS was calculated by Kaplan-Meier method, and Log-rank statistic were used to analyze differences between groups. Cox proportional hazard model for multivariate analysis were performed to evaluate the relationship between other clinical or pathological factors and prognosis. Characteristics included into multivariate analysis were chosen before data collection to be clinically significant. Differences were considered as statistically significant if P<0.05. All statistical analyses were performed by SPSS software, standard version 25.0 (IBM Corp., Armonk, NY, USA).

## Results

A total of 357 women were enrolled in our study, 247 (69.2%) patients were LVSI-, 110(30.8%) patients were LVSI+ (Table 1). Between LVSI+ and LVSI- group, a significant difference was found in BMI (*p* = 0.030), SCC value ≥1.5 ng/mg (*p* = 0.000), tumor size ≥2 cm (*p* = 0.002), FIGO stage (*p* = 0.000), lymph node involvement (*p* = 0.000) and depth of stromal invasion (*p* = 0.000). While difference in age, surgical approach and tumor differentiation were not significant. None of the 357 women presented parametrial invasion or positive surgical margin. Furthermore, binary Logistic regression indicated that among analyzed characteristics, depth of stromal invasion was the single independent risk factor of LVSI (Table 2). Both middle 1/3 invasion and deep 1/3 invasion can increase the risk of LVSI+ significantly, with a odds ratio (OR) of 3.494 (*p* = 0.000) and 15.203 (*p* = 0.000) separately.

**Table 1:** Demographic and clinical-pathological characteristic characteristics according to LVSI

Characteristics	LVSI-	LVSI+	*P* value
Age (years)			
< 45	112	41	.166
≥ 45	135	69	
BMI	23.15(2.94)	23.97(3.32)	.030
SCC (ng/ml)			
< 1.5	157	47	.000
≥ 1.5	90	63	
Tumor size			
< 2 cm	177	60	.002
≥ 2 cm	70	50	
FIGO stage			
IB1	217	78	.000
IIA1	30	32	
Surgical approach			
Laparotomy	24	8	.508
Laparoscopic	174	75	
Robotic	49	27	
Histopathologic grades			
G1	18	13	.259
G2	216	89	
G3	13	8	
Lymph node involvement			
Absent	243	97	.000
Present	4	13	
Stromal invasion			
Superficial 1/3	185	36	.000
Middle 1/3	56	48	
Deep 1/3	6	26	

Values are presented as mean (standard deviation) or number

Chi-square test and t-test for *P* values

*SCC* Squamous cell carcinoma antigen, *G1* Well differentiated, *G2* Moderately differentiated, *G3* Poorly or undifferentiated

**Table 2:** Multivariate analysis of clinical-pathological characteristics and lymphovascular space invasion

	*P* value	OR	95% CI of OR	
			Lower limit	Upper limit
Age				
< 45 years		Ref.		
≥ 45 years	.998	1.001	.558	1.796
BMI	.328	1.048	.954	1.151
Tumor size				
< 2 cm		Ref.		
≥ 2 cm	.615	1.169	.635	2.151
SCC				
< 1.5		Ref.		
≥ 1.5	.346	1.335	.732	2.434
Surgical approach				
Laparotomy	.407	Ref.		
Laparoscopic	.614	1.288	.481	3.449
Robotic	.247	1.937	.632	5.938
FIGO stage				
IB1		Ref.		
IIA1	.129	1.793	.843	3.812
Depth of stromal invasion				
Superficial 1/3	.000	Ref.		
Middle 1/3	.000	3.494	1.900	6.426
Deep 1/3	.000	15.203	4.668	49.516
Lymph node involvement				
Absent		Ref.		
Present	.085	3.443	.843	14.070
Histopathologic grades				
G1	.669	Ref.		
G2	.418	.669	.253	1.769
G3	.881	.892	.200	3.978

Binary Logistic regression for *P* values

*OR* Odds ratio, *CI* Confidence interval, *SCC* Squamous cell carcinoma antigen, *G1* Well differentiated, *G2* Moderately differentiated, *G3* Poorly or undifferentiated

Among 110 LVSI+ patients, 73 patients have received chemoradiotherapy after surgery, 31 patients received chemotherapy alone, 6 had no further treatment, and none received radiotherapy alone. Because of limited data size of other groups, only patients of chemoradiotherapy and chemotherapy were included into further analysis.

Demographic characteristics and clinicopathologic characteristics of LVSI+ patients received CT and CRT are showed in Table 3. Demographic characteristics such as age and BMI between chemotherapy group and chemoradiotherapy group were well balanced. All these patients have undergone a radical hysterectomy and pelvic lymphadenectomy with or without oophorosalpingectomy. None of these patients showed parametrial invasion or positive surgical margin. For included patients, FIGO Stage (2009), SCC value, surgical approach, tumor differentiation and lymph node metastasis were not significantly different. While deep stromal invasion presented a statistical significance (*P* = 0.001).

**Table 3:** Comparation of clinical-pathological characteristics of LVSI+ patients received CT and CRT

Characteristics	Chemotherapy	Chemoradiotherapy	*P* value
Age (years)			
< 45	14	25	.293
≥ 45	17	48	
BMI	24.09(3.26)	23.97(3.76)	.872
Tumor size			
< 2 cm	17	40	.997
≥ 2 cm	14	33	
FIGO stage			
IB1	21	48	.844
IIA1	10	25	
SCC (ng/ml)			
< 1.5	12	30	.821
≥ 1.5	19	43	
Surgical approach			
Laparotomy	3	4	.391
Laparoscopic	23	49	
Robotic	5	20	
Histopathologic grades			
G1	6	7	.346
G2	21	58	
G3	4	8	
Lymph node involvement			
Absent	29	60	.221
Present	2	13	
Stromal invasion			
Inner 1/3	18	15	.001
Middle 1/3	9	35	
Outer1/3	4	23	

Values are presented as mean (standard deviation) or number

Chi-square test and t-test for *P* values. Among 110 LVSI+ patients, 73 patients have received chemoradiotherapy, 31 received chemotherapy, 6 had no further treatment, and none received radiotherapy alone. Because of limited data size of other groups, only patients of chemoradiotherapy and chemotherapy were included

*SCC* Squamous cell carcinoma antigen, *G1* Well differentiated, *G2* Moderately differentiated, *G3* Poorly or undifferentiated

For all patients of chemotherapy group and chemoradiotherapy group, median follow-up time was 49.50 months (IQR 41.25–62.50) at the time of the analysis and 27.88% of patients had reached 5 years of follow-up. Up to the time of telephone interview, 9 patients were found relapsed (5 in chemotherapy group, 3 in chemoradiotherapy group and 1 in observation group). Among these patients, there were 2 pelvic recurrences, 2 pulmonary metastasis and 1 hepatic metastasis as well as skeletal metastasis in chemotherapy group; 2 multiple lymph node metastases throughout the body and 1 relapse in anal area in chemoradiotherapy group; 1 pelvic recurrence in observation group.

**Fig. 1:**
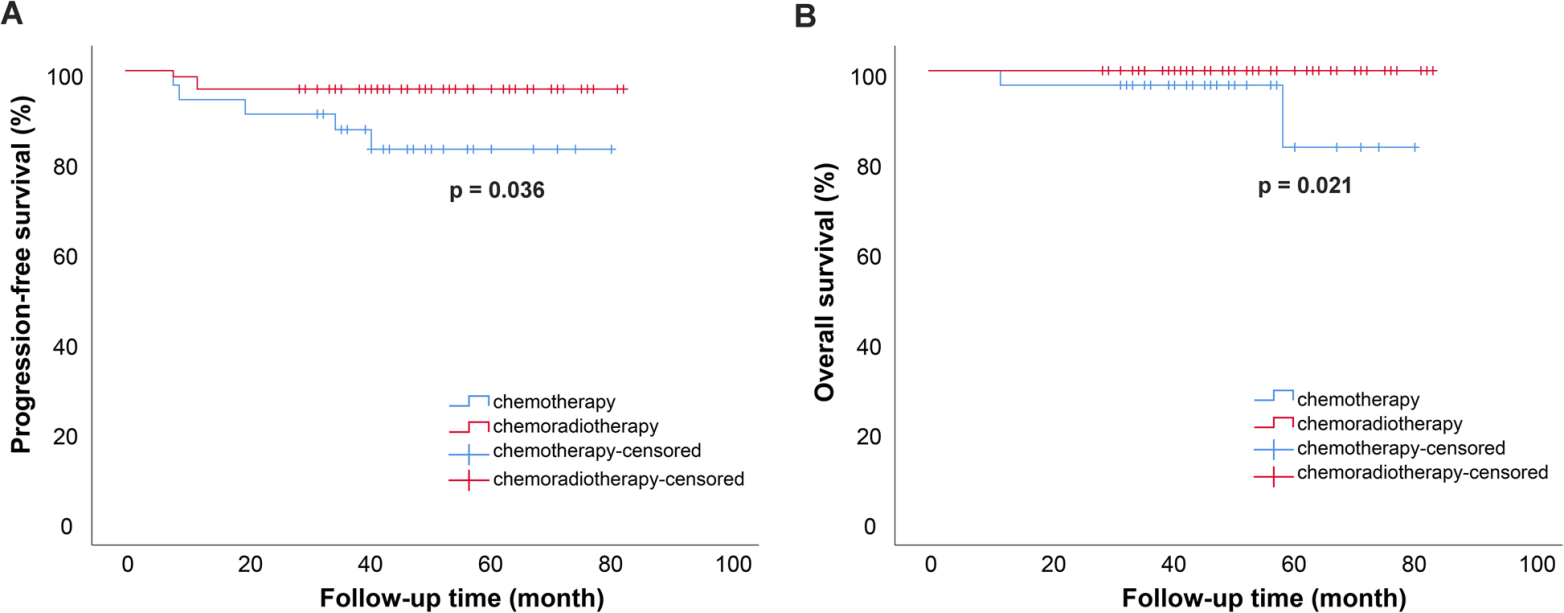
Survival curves based on postoperative therapy on LVSI+ patients (*n* = 104). Log-rank test for *p*-values. Survival curves are shown for **A** PFS and **B** OS. PFS, progression free survival; OS, overall survival

In univariate analysis of different characteristics and progression-free survival, the present study revealed that tumor differentiation (*P* = 0.001) and postoperative therapy (*P* = 0.036) had a significant influence on disease recurrence (Table 4). Between different postoperative therapies, CT group presented 5 relapses, with a mean PFS of 71.020 month, whereas 3 recurrences were presented at CRT group, with a mean PFS of 80.027 month (*P* = 0.036) (Fig. 1A). Other clinical- and pathological factors such as age, tumor size, SCC value, FIGO stage, DSI and lymph node invasion didn’t influent PFS significantly. As for surgical approach, we only conducted a comparison between laparoscopic surgery and robot-assisted surgery because of no recurrence in laparotomy group, and no significant difference were found. In multivariate analysis, Cox proportional hazard model showed that lymph node metastasis (*P* = 0.017), tumor differentiation (*P* = 0.036) and postoperative therapy (*P* = 0.021) can increase the risk of tumor relapse (Table 5).

**Table 4:** Univariate analysis of different characteristics and progression-free survival of LVSI+ population

	Mean PFS	SE	95% CI of PFS		P
			Lower limit	Upper limit	
Age					
< 45 years	77.564	3.025	71.635	83.493	.997
≥ 45 years	72.486	1.966	68.633	76.339	
Tumor size					
< 2 cm	76.985	2.594	71.901	82.068	.656
≥ 2 cm	72.368	2.048	68.354	76.381	
SCC (ng/ml)					
< 1.5	75.167	1.985	71.276	79.059	.377
≥ 1.5	76.451	2.560	71.434	81.468	
Surgical approach					
Laparotomy	–	–	–	–	–
Laparoscopic	78.345	2.422	73.597	83.092	.418
Robotic	65.120	1.842	61.510	68.730	
FIGO stage					
IB1	80.089	1.665	76.825	83.353	.072
IIA1	71.688	3.877	64.090	79.287	
Depth of stromal invasion					
Superficial 1/3	66.870	3.821	59.381	74.359	.153
Middle 1/3	80.152	1.985	76.262	84.042	
Deep 1/3	69.778	2.181	65.504	74.052	
Lymph node invasion					
Absent	79.135	1.691	75.820	82.450	.061
Present	62.028	5.345	51.551	72.505	
Histopathologic grades					
G1	77.000	3.795	69.562	84.438	.001
G2	80.354	1.502	77.410	83.299	
G3	49.667	7.237	35.481	63.852	
Adjuvant therapy					
Chemotherapy	71.020	4.136	62.913	79.127	.036
Chemoradiotherapy	80.027	1.681	76.732	83.323	

Log-rank test for *p*-values

*SE* Standard error, *CI* Confidence interval, *SCC* Squamous cell carcinoma antigen, *G1* Well differentiated, *G2* Moderately differentiated, *G3* Poorly or undifferentiated

**Table 5:** Multivariate analysis of different characteristics and progression-free survival of LVSI+ population

Characteristics	*P* value	HR	95% CI of HR	
			Lower limit	Upper limit
Tumor size				
< 2 cm		Ref.		
≥ 2 cm	.076	.065	.003	1.327
Surgical approach				
Laparotomy	.706	Ref.		
Laparoscopic	.992	.000	.000	.000
Robotic	.404	3.750	.168	83.764
FIGO stage				
IB1		Ref.		
IIA1	.123	8.073	.567	115.040
Depth of stromal invasion				
Superficial 1/3	.699	Ref.		
Middle 1/3	.642	.628	.089	4.446
Deep 1/3	.588	2.283	.115	45.388
Lymph node metastasis				
Absent		Ref.		
Present	.017	96.283	2.247	4125.335
Adjuvant therapy				
Chemotherapy		Ref.		
Chemoradiotherapy	.021	.042	.003	.621
Histopathologic grades				
G1	.022	Ref.		
G2	.540	2.722	.110	67.169
G3	.036	83.727	1.333	5260.175

A Cox proportional hazard regression model for *p*-values

*SE* Standard error, *HR* Hazard ratio, *CI* Confidence interval, *SCC* Squamous cell carcinoma antigen, *G1* Well differentiated, *G2* Moderately differentiated, *G3* Poorly or undifferentiated

Meanwhile, 3 deaths were identified in the present study, 2 in chemotherapy group and 1 in observation group. The 2 deaths of chemotherapy group occurred at 12th month after hepatic and skeletal metastasis and 59th month after pelvic recurrence separately. While up to the time of data analysis, all patients received chemoradiotherapy were alive. As a result of data limitation, we only reached to compared the overall survival between different adjuvant treatment regimens, and have found a statistical significance between CT and CRT group (*P* = 0.021) (Fig. 1B).

## Discussion

The current study revealed that deep stromal invasion was the single independent risk factor of LVSI+. In survival analysis, univariate analysis indicated that poorer tumor differentiation and postoperative chemotherapy alone are associated with higher possibility of recurrence for LVSI+ early-stage invasive squamous cervical carcinoma. Cox proportional hazard model indicated that the presence of lymph node metastasis, poorly or undifferentiated tumor and postoperative chemotherapy alone are associated with higher recurrence rate.

The present study found that DSI can significantly increase the incidence of LVSI+ in cervical cancer, which is consistent with previous evidences [19, 20]. Criteria of DSI varies from different studies, the current study divided depth of stromal invasion as superficial 1/3, middle 1/3, and deep 1/3, which is in accordance with the Sedlis criteria. Moreover, the present study showed that both middle 1/3 invasion and deep 1/3 invasion can significantly increase the incidence of LVSI+, validating the relationship between DSI and LVSI in spite of different definition of DSI. While the present study revealed that DSI is the only independent risk factor of LVSI+, more reports showed that lymph node metastasis is closely associated with LVSI+ [19–21]. Our study also found significant relationship between lymph node metastasis and LVSI+ in univariate analysis. In addition, since the publication of Laparoscopic Approach to Cervical Cancer (LACC) trial in 2018 [22], there have been controversy on optimal surgical approach of early-stage cervical cancer. It is believed that the utilization of manipulator allows malignant cells to spread into vascular and lymphatic vessels by squeezing tumor lesion, thus result in tumor relapse of pelvic cavity. Nevertheless, in the current study, different surgical approach didn’t influence the incidence of LVSI significantly. A recent prospective study also found that surgical approach and the utilization of manipulator are not associated with LVSI in cervical cancer [23].

To our knowledge, the present study is the first study focusing on postoperative treatment regimen of LVSI+ early-stage invasive squamous cervical carcinoma as well as the first study comparing postoperative chemotherapy alone and chemoradiotherapy in intermediate risk individuals of cervical cancer. In the current study, both univariate analysis and Cox proportional hazard model indicated that compared to postoperative CT alone, CRT is associated with prolonged PFS of cervical cancer. Being one of intermediate risk factors of poor prognosis, it is still controversial whether LVSI is an independent risk factor of higher recurrence rate and mortality. Nevertheless, when it is combined with other intermediate risk factors such as DSI and large tumor size, the recurrence rate increases up to 15–20% [17]. Cancer cells spread into vascular and lymphatic vessels might contribute to tumor involvement [19]. Therefore, LVSI might play a crucial role in metastasis. Based on its natural feature of involving inside vascular and lymphatic vessels, it is reasonable to consume that adjuvant systemic treatment contributes to inhibit cancer cell spread. Furthermore, as a result to limited access to effective radiotherapy, radiotherapy is not available for patients in many developing countries [3, 24]. At present, there are only limited data on the role of chemotherapy in postoperative treatment. A retrospective study comparing CT and RT in FIGO stage IB1 and IIA1 cervical carcinoma indicates that RT group had a significantly longer PFS, while difference in OS was not significant [24]. In a nation-wide study analyzed the prognosis of 555 intermediate-risk individuals with stage IB cervical cancer after different adjuvant therapies (chemotherapy, radiotherapy and chemoradiotherapy), and have found that patients who received postoperative chemotherapy alone presented similar PFS and OS with other two groups, suggesting the effectiveness of chemotherapy as adjuvant therapy in intermediate-risk cervical cancer [25]. However, existed studies are mostly retrospective analysis, to have a better understanding of optimal adjuvant treatment in LVSI+ early invasive cervical cancer, large-scale prospective trial is still warranted.

Survival analysis in the present study suggested that poorly or undifferentiated tumor is associated with higher risk of recurrence for cervical cancer. This is consistent with previous reports. A retrospective observational study analyzing 31,536 women with squamous cervical cancer revealed that compared to well-differentiated tumors, both moderately-differentiated tumors and poorly or undifferentiated tumors were independently related to decreased cause-specific survival. Furthermore, poorly or undifferentiated tumor s were associated with significantly decreased cause-specific survival compared to grade moderately-differentiated [26]. However, this report didn’t describe data on tumor recurrence. There have been studies on the mechanism for the relationship between tumor differentiation and prognosis in squamous cervical cancer. According to prior studies, poorly or undifferentiated tumors are associated with lower keratin, which is usually more aggressive with epithelial-mesenchymal transition (EMT) [27]. Epithelial cancer cells obtain migration and invasion ability in the bloodstream after EMT [28], leading to cancer cells spread and inferior prognosis. It is speculated that poorly or undifferentiated tumor alters prognosis through lower keratin [26]. Nevertheless, further investigation is still required to validate this theory.

The present study is a retrospective study, there might be confounding variables that were not taken into account. For example, baseline characteristic comparation of chemotherapy and chemoradiotherapy group indicated a statistical significance on depth of stromal invasion. In consideration of potential worse prognosis in women with DSI, chemoradiotherapy are more likely to be administered to this group of patients, thus weaken the impact of DSI on DFS and OS. A limitation of the study is that data size was limited. As a result, patients who received radiotherapy and only follow-up was not included in data analysis, which prevented us from organizing a comprehensive study of different adjuvant therapy on FIGO stages IB1 and IIA1 cervical squamous cancer patients with LVSI. However, in the real-world setting, some patients with intermediate pathologic risk factors are receiving postoperative radiotherapy alone or observation. In addition, no death was found in CRT group, hence sub-group analysis and multivariable analysis of OS were not available. Although considering the synergistic effects of chemotherapy and radiotherapy, it was reasonable to consume that OS of patients who received chemoradiation would be better than those who received chemotherapy in LVSI positive patients, clinical evidences are still required to confirm it. Therefore, large-scale studies are warranted in order to obtain a better understanding of adjuvant therapy on cervical cancer patients with intermediate risk. A weakness of this study is that toxicity and side effects of the regimens were not accounted for in this study, which might affect compliance and tolerance of patients. In addition to disease control, for patients with malignancy, quality of life also worth to discuss.

In spite of these limitations, this is the first study of postoperative treatment for LVSI+ early-stage invasive squamous cervical carcinoma. It is also the first study comparing postoperative chemotherapy alone and chemoradiotherapy in intermediate risk individuals of cervical cancer. Compared with existed studies, the follow-up time of the present study is relatively long. The current study revealed that DSI was significantly associated to LVSI+ for FIGO stages IB1 and IIA1 cervical squamous cancer patients. Compared with chemotherapy, chemoradiotherapy seems to improve progression-free survival in squamous cervical cancer patients with lymphovascular space invasion.

## Data Availability

The datasets used and analysed during the current study are available from the corresponding author on reasonable request.

## Abbreviations

CI: Confidence interval
CRT: Chemoradiotherapy
CT: Chemotherapy alone
DSI: Deep stromal invasion
EMT: Epithelial-mesenchymal transition
G1: Well differentiated
G2: Moderately differentiated
G3: Poorly or undifferentiated
HR: Hazard ratio
LVSI: Lymphovascular space invasion
OR: Odds ratio
OS: Overall survival
PFS: Progression-free survival
RT: Radiotherapy alone
SCC: Squamous cell carcinoma antigen
SE: Standard error

## References

[1] Sung H, Ferlay J, Siegel RL, Laversanne M, Soerjomataram I, Jemal A, Bray F (2021). Global cancer statistics 2020: GLOBOCAN estimates of incidence and mortality worldwide for 36 cancers in 185 countries. CA Cancer J Clin.

[2] Bhatla N, Aoki D, Sharma DN, Sankaranarayanan R (2018). Cancer of the cervix uteri. Int J Gynaecol Obstet.

[3] Barton MB, Frommer M, Shafiq J (2006). Role of radiotherapy in cancer control in low-income and middle-income countries. Lancet Oncol.

[4] dos Reis R, Burzawa JK, Tsunoda AT, Hosaka M, Frumovitz M, Westin SN, Munsell MF, Ramirez PT (2015). Lymphovascular space invasion portends poor prognosis in low-risk endometrial cancer. Int J Gynecol Cancer.

[5] Ayhan A, Sahin H, Sari ME, Yalcin I, Haberal A, Meydanli MM (2019). Prognostic significance of lymphovascular space invasion in low-risk endometrial cancer. Int J Gynecol Cancer.

[6] Hahn HS, Lee IH, Kim TJ, Lee KH, Shim JU, Kim JW, Lim KT (2013). Lymphovascular space invasion is highly associated with lymph node metastasis and recurrence in endometrial cancer. Aust N Z J Obstet Gynaecol.

[7] Hanson MB, van Nagell JR, Powell DE, Donaldson ES, Gallion H, Merhige M, Pavlik EJ (1985). The prognostic significance of lymph-vascular space invasion in stage I endometrial cancer. Cancer.

[8] Matsuo K, Garcia-Sayre J, Medeiros F, Casabar JK, Machida H, Moeini A, Roman LD (2015). Impact of depth and extent of lymphovascular space invasion on lymph node metastasis and recurrence patterns in endometrial cancer. J Surg Oncol.

[9] Pavlakis K, Rodolakis A, Vagios S, Voulgaris Z, Messini I, Yiannou P, Vlachos A, Panoskaltsis T (2017). Identifiable risk factors for lymph node metastases in grade 1 endometrial carcinoma. Int J Gynecol Cancer.

[10] Veade AE, Foote J, Ehrisman J, Broadwater G, Davidson BA, Lee PS, Secord AA, Berchuck A, Havrilesky LJ (2019). Associations between lymphovascular space invasion, nodal recurrence, and survival in patients with surgical stage I endometrioid endometrial adenocarcinoma. World J Surg Oncol.

[11] Rotman M, Sedlis A, Piedmonte MR, Bundy B, Lentz SS, Muderspach LI, Zaino RJ (2006). A phase III randomized trial of postoperative pelvic irradiation in stage IB cervical carcinoma with poor prognostic features: follow-up of a gynecologic oncology group study. Int J Radiat Oncol Biol Phys.

[12] Sedlis A, Bundy BN, Rotman MZ, Lentz SS, Muderspach LI, Zaino RJ (1999). A randomized trial of pelvic radiation therapy versus no further therapy in selected patients with stage IB carcinoma of the cervix after radical hysterectomy and pelvic lymphadenectomy: a gynecologic oncology group study. Gynecol Oncol.

[13] Ho CM, Chien TY, Huang SH, Wu CJ, Shih BY, Chang SC (2004). Multivariate analysis of the prognostic factors and outcomes in early cervical cancer patients undergoing radical hysterectomy. Gynecol Oncol.

[14] Rosa DD, Medeiros LR, Edelweiss MI, Pohlmann PR, Stein AT. Adjuvant platinum-based chemotherapy for early stage cervical cancer. Cochrane Database Syst Rev. 2012;13;6(6):CD005342. 10.1002/14651858.CD005342.pub3 PMC4164460 22696349

[15] Kim K, Kang SB, Chung HH, Kim JW, Park NH, Song YS (2009). Comparison of chemoradiation with radiation as postoperative adjuvant therapy in cervical cancer patients with intermediate-risk factors. Eur J Surg Oncol.

[16] Ryu SY, Park SI, Nam BH, Cho CK, Kim K, Kim BJ, Kim MH, Choi SC, Lee ED, Lee KH (2011). Is adjuvant chemoradiotherapy overtreatment in cervical cancer patients with intermediate risk factors?. Int J Radiat Oncol Biol Phys.

[17] Takekuma M, Kasamatsu Y, Kado N, Kuji S, Tanaka A, Takahashi N, Abe M, Hirashima Y (2017). The issues regarding postoperative adjuvant therapy and prognostic risk factors for patients with stage I-II cervical cancer: a review. J Obstet Gynaecol Res.

[18] Kim H, Park W, Kim YS, Kim YJ (2020). Chemoradiotherapy is not superior to radiotherapy alone after radical surgery for cervical cancer patients with intermediate-risk factor. J Gynecol Oncol.

[19] Yan W, Qiu S, Ding Y, Zhang Q, Si L, Lv S, Liu L (2019). Prognostic value of lymphovascular space invasion in patients with early stage cervical cancer in Jilin, China: a retrospective study. Medicine (Baltimore).

[20] Zhu J, Cao L, Wen H, Bi R, Wu X, Ke G (2020). The clinical and prognostic implication of deep stromal invasion in cervical cancer patients undergoing radical hysterectomy. J Cancer.

[21] Milam MR, Frumovitz M, dos Reis R, Broaddus RR, Bassett RL, Ramirez PT (2007). Preoperative lymph-vascular space invasion is associated with nodal metastases in women with early-stage cervical cancer. Gynecol Oncol.

[22] Ramirez PT, Frumovitz M, Pareja R, Lopez A, Vieira M, Ribeiro R, Buda A, Yan X, Shuzhong Y, Chetty N (2018). Minimally invasive versus abdominal radical hysterectomy for cervical cancer. N Engl J Med.

[23] Liu Y, Huang S, Ming X, Jing H, Li Z (2021). Surgical approach and use of uterine manipulator are not associated with LVSI in surgery for early-stage cervical cancer. J Minim Invasive Gynecol.

[24] Li L, Song X, Liu R, Li N, Zhang Y, Cheng Y, Chao H, Wang L (2016). Chemotherapy versus radiotherapy for FIGO stages IB1 and IIA1 cervical carcinoma patients with postoperative isolated deep stromal invasion: a retrospective study. BMC Cancer.

[25] Matsuo K, Shimada M, Yokota H, Satoh T, Katabuchi H, Kodama S, Sasaki H, Matsumura N, Mikami M, Sugiyama T (2017). Effectiveness of adjuvant systemic chemotherapy for intermediate-risk stage IB cervical cancer. Oncotarget.

[26] Matsuo K, Mandelbaum RS, Machida H, Purushotham S, Grubbs BH, Roman LD, Wright JD (2018). Association of tumor differentiation grade and survival of women with squamous cell carcinoma of the uterine cervix. J Gynecol Oncol.

[27] Cancer Genome Atlas Research N, Albert Einstein College of M, Analytical Biological S, Barretos Cancer H, Baylor College of M, Beckman Research Institute of City of H, Buck Institute for Research on A, Canada’s Michael Smith Genome Sciences C, Harvard Medical S, Helen FGCC (2017). Integrated genomic and molecular characterization of cervical cancer. Nature.

[28] Azevedo AS, Follain G, Patthabhiraman S, Harlepp S, Goetz JG (2015). Metastasis of circulating tumor cells: favorable soil or suitable biomechanics, or both?. Cell Adhes Migr.

